# Patterns of adult body mass in sub-Saharan Africa

**DOI:** 10.1080/16549716.2018.1556497

**Published:** 2019-01-17

**Authors:** Moffat J. Nyirenda, Michèle Ramsay, Peter Byass

**Affiliations:** London School of Hygiene and Tropical Medicine, London, UK; STIAS Wallenberg Research Centre, Stellenbosch University, Stellenbosch, South Africa; Sydney Brenner Institute for Molecular Bioscience, Faculty of Health Sciences, University of the Witwatersrand, Johannesburg, South Africa; Epidemiology and Global Health, Umeå University, Umeå, Sweden

Amidst rising global concerns around dietary and exercise patterns, obesity and non-communicable diseases, sub-Saharan Africa is once again trailing behind in terms of reliable population-based data, often being forced to fall back on modelled estimates [1]. Thus this Special Issue on body mass patterns in sub-Saharan Africa, based on large-scale primary data, is both appropriate and timely. The detailed methodology behind these studies is described separately [2].

The 40 to 60 year age group is particularly pertinent given that African populations are ageing and life expectancy at 60 years is increasing rapidly. Overall life expectancy in the World Health Organization (WHO) Africa Region rose by 10.3 years between 2000 and 2016, reaching 61.2 years [3]. Thus this 40 to 60 year cohort will carry forward current exposures into the older age groups of the future. The results here show very clearly that there are huge obesity differentials between women and men – up to eighteenfold  among women at one site – which means that the public health of obesity in Africa must always be disaggregated by sex.

The work in this Special Issue comes from a collaborative centre under the umbrella of the Human Heredity and Health in Africa Consortium (H3Africa), which aims to promote an understanding of genomic and environmental contributions to common traits in Africa by supporting studies led from the continent. The Africa Wits–INDEPTH Partnership for Genomic Studies (AWI-Gen Collaborative Centre) is one of six studies within H3Africa which has collected data relevant to obesity and several cardiovascular and metabolic traits [4]. By joining forces, these studies have established a joint vision and collaborative resource, referred to as the Cardiovascular H3Africa Innovation Resource (CHAIR), which aims to establish a large cohort of over 50,000 African participants from 13 African countries [5–7]. This effort is a unique African initiative that will include genome-wide genotyping for association studies and will provide opportunities to further explore the genetic contribution to obesity across different African settings.

Other important roles of H3Africa – funded by the National Institutes of Health (NIH) in the United States (US) and the Wellcome Trust in the United Kingdom (UK) – include contributing to the development of infrastructure for genomic research in Africa and enhancing capacity for researching both non-communicable and infectious diseases in an African context [5]. HIV, malaria and tuberculosis – in combination with the effects of clinical treatments and approaches to these epidemics in different African countries – contribute to body composition and at a population level will influence the prevalence and morbidity associated with obesity. The site-specific papers presented in this Special Issue [8–13] make important contributions to understanding some of the correlates with obesity in six communities in four African countries, highlighting the diversity of contributing factors and revealing some interesting regional differences. From a public health perspective it is clear that interventions to address the sequelae of increases in obesity in several African countries will require careful assessment and targeted strategies, informed by good quality data.

**Figure 1.**
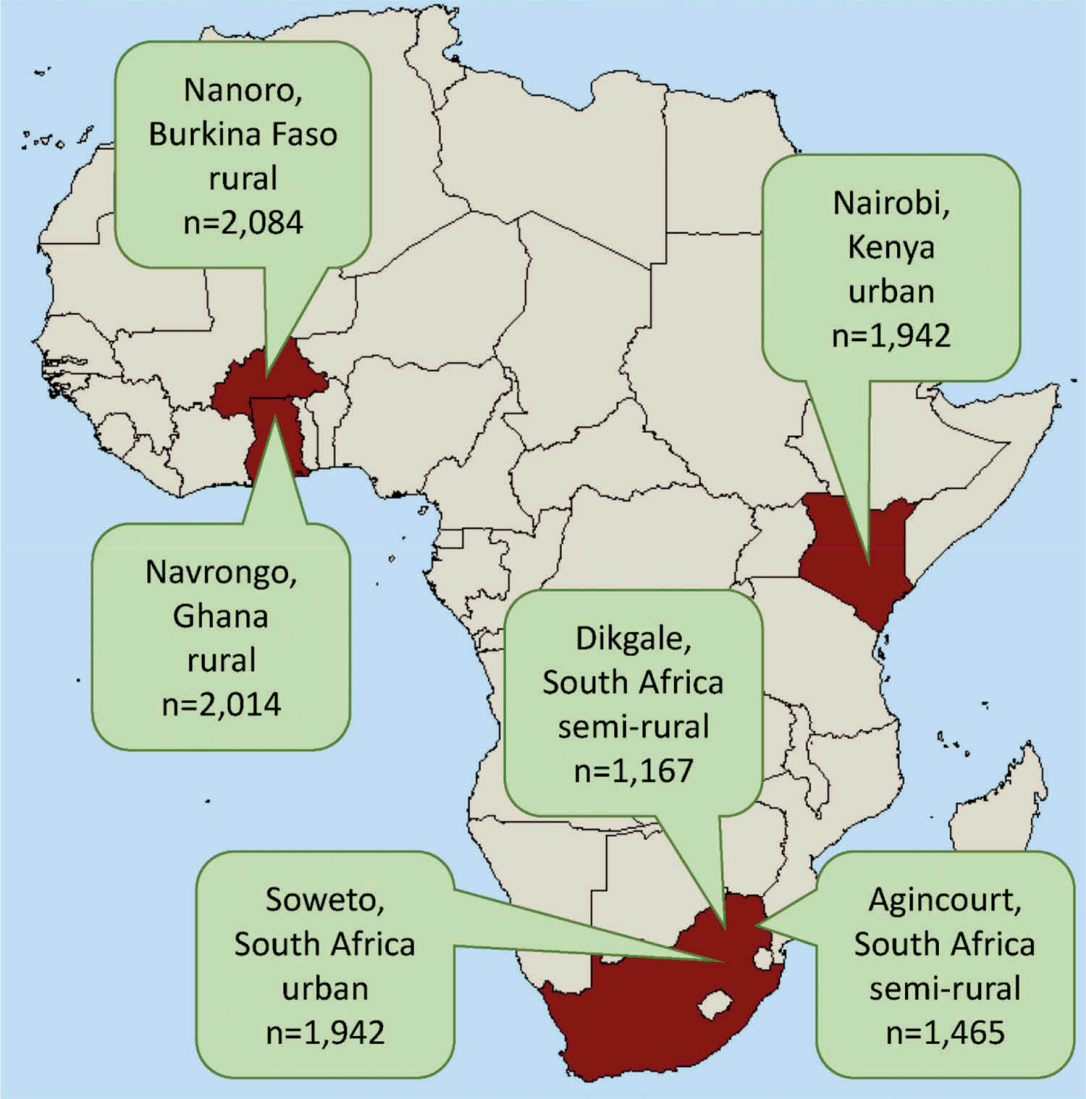
Details of the six sites in four countries contributing to the H3Africa AWI-Gen study.

The six AWI-Gen participating study sites, five of which are members of the INDEPTH Network [14], are located across Africa (Figure 1). They cover a range of living conditions and geographies. Three of the study sites (Navrongo, Nairobi and Agincourt) previously contributed to a standardised comparison of non-communicable disease mortality patterns [15]. While specific local population study sites cannot be demonstrated to be nationally representative, or indeed to be representative of anything beyond their boundaries, nevertheless piecing together a picture from specific well-studied populations across sub-Saharan Africa is a reasonable strategy in the absence of more comprehensive data [16]. In the overall AWI-Gen study, being able to link longitudinal socio-demographic, biomedical and genomic data within specific populations is a powerful advantage.

It is clear from the synthesis paper in this Special Issue [17] that patterns of body mass vary widely between study sites and by sex. The obesity levels were at less than 5% for both women and men at both West African sites, while the majority of women across the three South African sites were found to be obese. The main determinants of these variations in body mass index (BMI) across Africa and by sex remain to be fully explored.

This collection of studies provide useful common insights on BMI patterns across the continent. For example, whereas higher socio-economic and educational status are associated with lower BMI in high-income countries, in Africa these characteristics increase the risk of high BMI. Appropriate interventions will therefore require a nuanced understanding of the distribution of the underlying risk factors. What are the major determinants of inequalities in BMI trends between men and women? Are there sex-specific protective factors or behaviours in West Africa, or is it simply a matter of time before obesity levels also rise there?

These studies provide both valuable baseline data and a unique platform on which to build in order to understand patterns of BMI and their associations with disease in Africa. For example, the detailed characterisation or phenotyping of different populations will ensure that the planned genomic studies yield reliable data with huge potential for scientific discoveries.

Tackling non-communicable diseases in Africa effectively requires generating reliable local evidence that can inform appropriate local policies. Relatively little funding is available to stimulate and sustain such research on non-communicable diseases in Africa. In this respect, the H3Africa programme is unprecedented, championing high-quality research across the continent which is led by African scientists.

This is just the first step in the exploration of AWI-Gen data. A second wave of data and sample collection will enable a series of longitudinal cohorts that will provide an opportunity for studying changes in body composition and obesity – together with related behavioural and biological risk factors – in order to increase understanding of health transitions in Africa.
